# Assessing the Racial and Ethnic Disparities in Breast Cancer Mortality in the United States

**DOI:** 10.3390/ijerph14050486

**Published:** 2017-05-05

**Authors:** Clement G. Yedjou, Paul B. Tchounwou, Marinelle Payton, Lucio Miele, Duber D. Fonseca, Leroy Lowe, Richard A. Alo

**Affiliations:** 1 Natural Chemotherapeutics Research Laboratory, Research Centers in Minority Institutio (RCMI)-Center for Environmental Health, College of Science, Engineering and Technology, Jackson State University, 1400 Lynch Street, Box 18750, Jackson, MS 39217, USA; paul.b.tchounwou@jsums.edu (P.B.T.); gomezfonseca@gmail.com (D.D.F.); 2 Center of Excellence in Minority Health and Health Disparities, School of Public Health, Jackson State University, Jackson Medical Mall-Thad Cochran Center, 350 West Woodrow Wilson Avenue, Jackson, MS 39213, USA; marinelle.payton@jsums.edu; 3 Department of Genetics, LSU Health Sciences Center, School of Medicine, 533 Bolivar Street, Room 657, New Orleans, LA 70112, USA; lmiele@lsuhsc.edu; 4 Lancaster Environment Centre, Lancaster University, Bailrigg, Lancaster LA1 4YW, UK; leroy.lowe@gettingtoknowcancer.org; 5 Department of Civil and Environmental Engineering, College of Science, Engineering and Technology, Jackson State University, 1400 Lynch Street, Box 18750, Jackson, MS 39217, USA; richard.a.alo@jsums.edu

**Keywords:** breast cancer, racial disparity, health disparity, African American

## Abstract

Breast cancer is the second leading cause of cancer related deaths among women aged 40–55 in the United States and currently affects more than one in ten women worldwide. It is also one of the most diagnosed cancers in women both in wealthy and poor countries. Fortunately, the mortality rate from breast cancer has decreased in recent years due to increased emphasis on early detection and more effective treatments in White population. Although the mortality rates have declined in some ethnic populations, the overall cancer incidence among African American and Hispanic populations has continued to grow. The goal of the present review article was to highlight similarities and differences in breast cancer morbidity and mortality rates primarily among African American women compared to White women in the United States. To reach our goal, we conducted a search of articles in journals with a primary focus on minority health, and authors who had published articles on racial/ethnic disparity related to breast cancer patients. A systematic search of original research was conducted using MEDLINE, PUBMED and Google Scholar databases. We found that racial/ethnic disparities in breast cancer may be attributed to a large number of clinical and non-clinical risk factors including lack of medical coverage, barriers to early detection and screening, more advanced stage of disease at diagnosis among minorities, and unequal access to improvements in cancer treatment. Many African American women have frequent unknown or unstaged breast cancers than White women. These risk factors may explain the differences in breast cancer treatment and survival rate between African American women and White women. New strategies and approaches are needed to promote breast cancer prevention, improve survival rate, reduce breast cancer mortality, and ultimately improve the health outcomes of racial/ethnic minorities.

## 1. Introduction

Breast cancer is the second leading cause of cancer related deaths of women aged 40–55 in the United States and currently affects more than one in ten women worldwide [1]. In the United States African American and Hispanic women have a lower incidence of breast cancer compared to White women. Although African American and Hispanic women have a lower incidence of breast cancer, they have a higher mortality rate. A scientific data collected between 2000 and 2010 revealed that cancer incidence is higher among Black men compared to White men and higher among White women compared to Black women [2]. However, cancer mortality over the same time period was higher for both Black men and women compared to White men and women [3]. Scientific studies have identified possible differences in biological properties and especially in the plasma levels of growth factors and hormones [4], reproductive factors [5,6], susceptibility loci [7,8], and primary tumor characteristics, including the presence and expression of steroid and growth factor receptors [6,9,10,11], cell cycle proteins [12,13,14,15], tumor suppressor genes [15,16], and chromosomal abnormalities [17], between African American women and White women. These possible difference in biological properties between African American women and White women have the potential to influence breast cancer screening and treatment outcomes between the two ethnic groups. Since early 1990s, several strategies including early detection and diagnosis, reduction of tobacco smoking, widespread of cancer breast screening, and improvement of breast cancer therapies have been developed to improve the health of patients with breast cancer [18,19]. Although there has been an improvement in early detection, diagnosis, and screening, many Black women are less likely to get better treatment compared with White women [20,21]. Therefore, new strategies and approaches are needed to promote breast cancer prevention, improve survival rate, reduce breast cancer mortality, and ultimately improve the health outcomes of racial/ethnic minorities.

## 2. Methodology

A review of relevant scientific publications addressing breast cancer disparity and mortality rate primarily among African American women compared to White women in the United State was done using Google Scholar (https://scholar.google.com/). Protocols and initiatives by government programs and other non-governmental agencies were extensively studied, and archives of media coverage on breast cancer. Additionally MEDLINE, and PUBMED (https://www.ncbi.nlm.nih.gov/pubmed) searches were conducted. We mostly examined populations of White women and African American women in the United States. An attempt was made to include all relevant references examining the nature and magnitude as well as the major risk factors associated with breast cancer disparity.

## 3. Results and Discussion

The result and discussion sessions of this review article provide an overview of breast cancer, racial/ethnic disparities in breast cancer, breast cancer incidence and mortality rate linked to hereditary, major risk factors of breast cancer among minority population, breast cancer treatment and health disparity.

### 3.1. Overview of Breast Cancer

Breast cancer is a malignant tumor that starts in the cells of the breast. It is found mostly in women, but men can get breast cancer too. It is the most diagnosed cancer in women both in the developing and wealthy or developed countries. Worldwide, breast cancer survival rates vary greatly with approximately 80% in North America, Japan and Sweden to about 60% in middle-income countries and below 40% in poor countries [22]. African American women are more likely to be diagnosed with breast cancer at a younger age than White women [23]. For example, the median age at diagnosis for African American women is 59, compared to 63 for White women [23]. However, breast cancer incidence is lower among African-American women than among White women [23]. Although the incidence of breast cancer is slightly low among African American women, the mortality rate of breast cancer is significantly higher in this ethnic group compared to White women [24,25].

### 3.2. Racial/Ethnic Disparities in Breast Cancer

**Figure 1:**
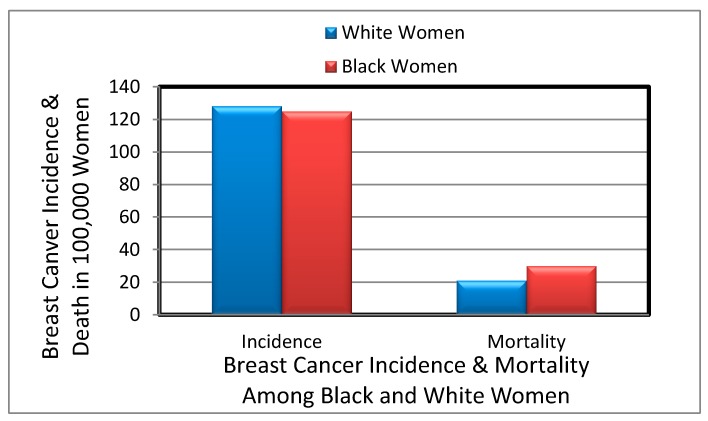
Number of breast cancer incidence and mortality among Black women and White women.

Racial/ethnicity disparities in breast cancer mortality rates are not completely understood, but the common risk factors include socioeconomic status, differential access to health care, and disease related molecular mechanistic differences [26]. There are a disproportionate number of cancer deaths occurring among African Americans with approximately 33% higher risk of dying of cancer than Whites. Recent report demonstrates that African American with breast cancer have a poorer prognosis compared to non-Hispanic Whites when diagnosed at a similar age and stage [27]. Overall, the mortality rate of breast cancer is still higher among Black, Hispanic, and Native American women than among White women [28,29]. Between 2002 and 2008, the relative 5 year survival rate for breast cancers incidence was 78% among Black women and 90% among White women [30]. The gap in breast cancer mortality rate between Black women and White women continues to increase. Between 2000 and 2010, the breast cancer mortality disparity ratio increased from 30.3% to 41.8% among African American women. At the advanced stage, 5% of breast cancers are detected among White women compared to 8% of breast cancers among Black women [30]. Number of breast cancer incidence and mortality per 100,000 women is represented in Figure 1 according to a 2015 report.

A recent collaborative epidemiological study reported that Black males have high incidence rates for all cancers combined (12% higher) and for the most common cancers, including cancers of the prostate, lung, colorectum, kidney, and pancreas. In contrast, Black females have a 6% lower overall incidence rate for all cancers combined and for many cancers, including lung, breast (only 3% lower), and uterine cancers [31]. Cancer incidence rates between Non-Hispanic (NH) Blacks and Whites in the United States between 2008 and 2012 are reported in Table 1 below.

### 3.3. Majors Risk Factors in Breast Cancer Affecting Minority Populations

The specific causes of breast cancer still remain unknown, but we do know that a woman’s chances of developing breast cancer are related to a wide range of risk factors including woman’s age, genetic factors, family history, personal health history, and diet. Lifestyle and environmental factors are also known to be responsible for a considerable portion of cancer incidence worldwide [32].

#### 3.3.1. Age and Sex Risk Factors in Breast Cancer

Age and sex are the most important risk factors to be considered in breast cancer incidence rates. Breast cancer incidence rates are higher among Blacks than Whites for women under age 45. The median age of diagnosis is 58 years for Black women compared with 62 years for White women [33]. The overall lifetime risk is 1 in 9 Black women expected to be diagnosed with breast cancer compared with 1 in 8 White women. Approximately 1% of all cases of breast cancer occur in men. At the age of up to 30 the risk of an individual developing breast cancer is 1 in 1900, but rises to 1 in 15 by the age of 70.

#### 3.3.2. Genetic Risk Factors in Breast Cancer

Family history has long been known to be a risk factor for breast cancer. For example, if members of a patient's family have had particular types of cancer, the patient will have an increased risk of developing breast cancer [34,35].

*BRCA1* and *BRCA2* are human genes that produce tumor suppressor proteins. These proteins help repair damaged DNA and, therefore, play a role in ensuring the stability of the cell’s genetic material. When either of these genes is mutated, altered, or does not function correctly, DNA damage may not be repaired properly. As a result, cells are more likely to develop additional genetic alterations that can lead to cancer development. The mutations of these two genes (*BRCA1* and *BRCA2*) are thought to account for between 5% and 10% of all breast cancer cases [36] and approximately 15% for ovarian cancers [37]. Female women with *BRCA1* gene have an increased risk of developing breast cancer at an early age [38].

**Figure 2:**
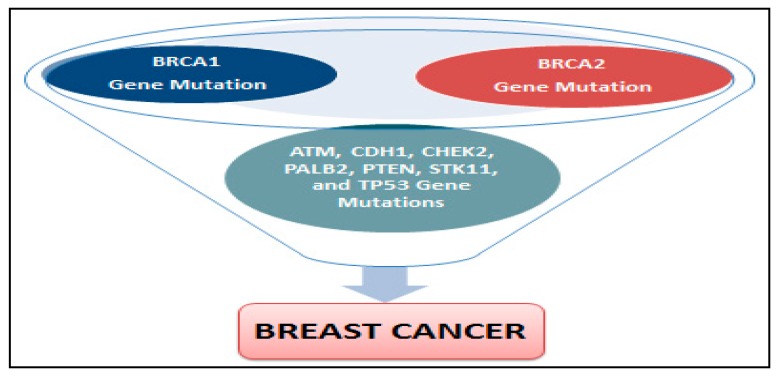
Possible genes mutations associated with breast cancer development.

A harmful *BRCA1* or *BRCA2* mutation can be inherited from a person’s mother or father. Each child of a parent who carries a mutation in one of these genes has a 50% chance to inherit the mutation. Specific inherited mutations in *BRCA1* and *BRCA2* increase the risk of female breast and ovarian cancers, and they have been associated with increased risks in many cancer types. Together, *BRCA1* and *BRCA2* mutations account for about 20 to 25% of hereditary breast cancers [39] and about 5 to 10% of all breast cancers [36]. In human breast cancer, genetic deletions have been demonstrated to be one of the major genetic abnormalities. Loss of heterozygosity in breast cancer has been documented at several chromosomal locations, including 1p, 1q, 2p, 3p, 6q, 7q, 8q, 9q, 11p, 11q, 13q, 15q, 16q, 17p, 17q, 18p, 18q and 22q [40]. The Loss of heterozygosity in few of chromosomal regions or locations have been shown to include a known 6 tumor suppressor gene implicated in breast cancer, such as *p53* at chromosome 17q13 [41,42], *BRCA-1* at 17q21 [43] and *BRCA-2* at 13q12-13 [44]. The differences in the genetics and biology of breast cancer incidence among Black women compared with White women are well documented in the literature. A breast cancer research that has evaluated 4885 White patients, 1,016 Black patients, and 777 Hispanic patients reported a significant differences in 5-year survival rates [45]. Finding from this research reported a five-year survival rate of 75 ± 1% for white patients, 70 ± 2% for Hispanic patients, and 65 ± 2% for black patients [45]. In addition to mutations in *BRCA1 and BRCA2* genes, mutations of *ATM, CDH1, CHEK2, PALB2, PTEN, STK11, TP53 (p53)* genes can also increase the risk of breast cancer [46]. Possible genes mutations associated with breast cancer development are reported in Figure 2.

Despite most of the breast cancers are not of hereditary origin, lifestyle and environmental factors such as diet, obesity, smoking, alcohol consumption, infection diseases, and radiation have a profound influence on cancer development [47]. Although the hereditary factors cannot be modified, lifestyle and environmental factors that affect the incidence and mortality rate of breast cancer are modifiable and can be prevented.

#### 3.3.3. Lack of Physical Activity Risk Factors in Breast Cancer

Breast cancer has been one of the most extensively studied cancers in relation to physical activity, with more than 60 studies that have reported this association published in Asia, and Australia, Europe, and United State. Many studies in the United States and around the world have consistently found that physically active women have a lower risk of developing breast cancer compared to inactive women. However, the percentage of risk reduction achieved through physical exercise varies between 20% and 80% with the greatest risk reduction seen among those who are most active [48,49]. Similarly, a review of 73 observational studies indicated that moderate to vigorous physical activity reduces breast cancer risk by an average of 25% in pre- and post-menopausal women compared with inactive women [50]. Although it is possible that women who are physically active experience a reduction in risk of breast cancer, the biological mechanisms of action explaining this association are still largely unknown. A 2006 report indicated that physical activity may prevent against breast cancer and tumor development by lowering hormone levels, reducing levels of insulin and insulin-like growth factor I, improving the immune response; and assisting with weight maintenance to avoid a high body mass and excess body fat [48].

#### 3.3.4. Poor Diet, Obesity, and alcohol Consumption Risk Factors in Breast Cancer

A systematic review conducted by Albuquerque and his collaborators found that Mediterranean dietary pattern and diets rich of vegetables, fruit, fish, and soy are associated with a decreased risk of breast cancer [51]. Other studies reported that intakes of dietary fiber, fruit, and vegetables significantly reduce breast cancer risk factors [52,53,54,55]. However, poor diet and lack of physical exercise may lead to weight gain which plays a role in the development of breast cancer and survival rate of African American women [56].

**Figure 3:**
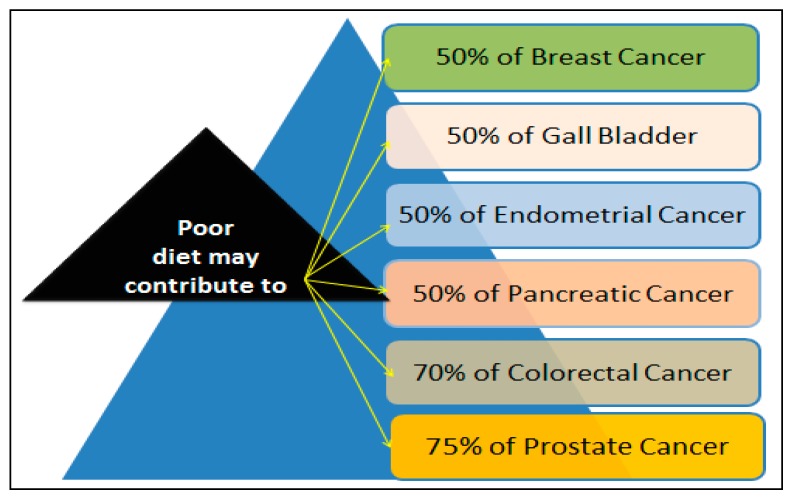
High percentages of breast cancer and other cancer deaths associated with poor diet.

A recent report has linked obesity to an increased risk of developing breast cancer [57]. Other report indicated that obesity increases the risk of breast cancer in postmenopausal women by approximately 30% for women with a Body Mass Index (BMI) of greater than 31 kg/m^2^. A BMI of greater than 35 kg/m^2^ is associated with a doubling of the risk of breast cancer [58]. How diet contributes to cancer remains likely unknown. A 2000 report indicated that diet may contribute to approximately 50% of all to breast cancers [59]. In addition to breast cancer, high percentages of other cancers that have been linked to diet factors are represented in Figure 3.

Alcohol consumption plays an important role in the development of breast cancer. For example, previous studies have suggested that increased breast cancer risk is associated with both the amount and the duration of alcohol consumption [60,61]. Nagata et al. (1997) [62] provided evidence that alcohol consumption may increase serum levels of estradiol, suggesting that alcohol consumption is indirectly associated with the development of breast cancer by increasing the exposure to estrogen. A collaborative re-analysis of individual data from 53 epidemiological studies, including 58,515 women with breast cancer and 95,067 women without breast cancer has evaluated the association between alcohol consumption and breast cancer incidence [63]. They found a linear increase in breast cancer risk with increasing alcohol consumption. They concluded that the relative risk of breast cancer is 1.32 for women drinking between 35 g and 44 g of alcohol per day and 1.46 for women drinking 45 or more grams of alcohol per day. They also found a 7.1% increase in the relative risk of breast cancer for 8 each additional 10 g of alcohol consumed per day. The summary from these epidemiological studies estimated that 4% of all breast cancers are attributable to alcohol consumption [63]. The International Agency for Research on Cancer estimates that approximately one fourth to one third of cancer cases are associated with elevated body weight and inadequate physical activity [64]. Previous scientific reports indicated that African Americans are more overweight, obese, and have higher BMI and waist-to-hip ratios compared to Caucasians [65,66]. It is estimated that more than 50% of African American women aged 40 years or older are obese and more than 80% are overweight [67]. The lack of physical exercise may explain why African American women have higher rates of obesity, a major risk factor of breast cancer in Black women compared to other ethnic groups [68].

### 3.4. Breast Cancer Prevention, Treatment and Health Disparity

A recent report suggests that half of breast cancer cases may be prevented if chemoprevention is applied in appropriate at-risk populations and the major modifiable risk factors such as achieving and maintaining a healthy weight, regular physical activity, and minimal alcohol intake are instituted [69]. However, scientific evidence revealed that racial/ethnic disparities in breast cancer are attributed to risk factors such as lack of medical coverage, barriers to early detection and screening, more advanced stage of disease at diagnosis among minorities, and unequal access to improvements in cancer treatment. These factors may explain the differences in survival rate between African American and White women [70,71,72]. There is also scientific evidences suggesting that the prevalence of estrogen receptor-positive breast tumors may be lower in African Americans and Hispanics than in Whites [46,73], which might account for racial/ethnic differences in the use of tamoxifen. Further evidence suggests that, because of the increased risk of stroke, pulmonary embolism, and deep vein thrombosis associated with tamoxifen, African Americans, who already have a higher prevalence of risk factors for these conditions, may receive less overall benefit from tamoxifen [27].

National Cancer Institute (NCI)-supported research indicated that aggressive breast tumors are more common in younger African American and Hispanic women living in low socioeconomic status (SES) areas. This more aggressive form of breast cancer is less responsive to standard cancer treatments and is associated with lower survival rate [72]. A 2000 Survey of eight New York State hospitals found that physicians have more negative perceptions of African Americans and persons of low or middle SES than of Whites and persons of high socioeconomic status [74]. This finding and lack of information on how physician attitudes toward patients affect their care need further research, particularly with regard to how such negative perceptions might contribute to racial/ethnic disparities in cancer treatment.

A 2014 study by the Carolina Breast Cancer found racial differences in physical activity among breast cancer survivors revealed that African American, compared to Caucasian women, are significantly less likely to meet national physical activity guidelines after diagnosis [75]. The lack or limited physical exercises have some implications for breast cancer care. The Sisters Network Inc. suggests that only 47% of African American Breast Cancer survivors may be meeting these physical activity guidelines. Another study by the Northeast Ohio Breast Cancer survivors found a gradual decline in physical activity levels after high school completion in African American compared to White women and revealed that only 12.3% of African American breast cancer survivors were meeting exercise guidelines [76]. A proposed breast cancer care model recommended that breast cancer patients should be educated about the importance of physical exercise at the point of breast cancer diagnosis, and provide them with the necessary support to stay active during the stage of breast cancer diagnosis-treatment continuum and beyond [77].

### 3.5. Mechanisms of Apoptosis Resistance in Breast Cancer Treatment

Apoptosis resistance in breast cancer is the major cause for female cancer mortality in the wealthy countries. Understanding apoptosis in disease conditions is very important as it not only gives insights into the pathogenesis of a disease, but may also give clues on how the disease can be treated. In cancer, there is a loss of balance between cell division and cell death and cells that should have died, but did not receive the signals to do so. The problem can arise in any one step along the way of apoptosis. Several tumor suppresser genes have been identified, and mutations in these genes have been associated with a variety of neoplastic conditions. One example is the downregulation of p53, a tumor suppressor gene, which results in reduced apoptosis and enhanced tumor growth and development [78]. Inactivation of p53 has been linked to many human cancers regardless of the mechanism [79,80]. It is an important regulator of cell cycle progression and DNA damage repair. Many studies have reported that p53 is mutated in most African American breast cancer [12,13,46], but other studies have not [81]. The tumor suppressor gene *RASSF1A* modulates multiple apoptotic and cell cycle checkpoint pathways, restricting exit from G1 [82]. *RASSF1A* in breast cancer among women aged <50 years was found to be methylated in 76% of cases of African American women versus 29% of cases of Caucasian women (*p* < 0.0001) [15]. *RARβ* (trend, *p* = 0.01) and *HIN-1* (*p* < 0.0001) are more often methylated in the tumors of African American women [15]. RARβ causes cell cycle arrest, growth inhibition and induction of apoptosis, and HIN-1 in cycle reentry [83]. Scientific data indicate that breast cancer in African American women is characterized by enhanced expression of cyclin E [12,13,84], which regulates entry into S-phase. High levels of cyclin E expression are associated with larger, estrogen receptor (ER)-negative tumors, consistent with tumors in African American women [85].

Despite an increased emphasis on early detection/screening, more effective treatments, and the greater understanding of the molecular pathways underlying breast cancer biology, a major fraction of patients have recurrent disease that becomes refractory to most of the available therapies [86]. Current treatment depends on the type and the stage which can be local or systemic. Local treatment involves the treatment without affecting the rest of the body. It includes: (1) surgery which may be breast-conserving surgery whereby part of the breast is removed and mastectomy whereby the entire breast is removed; (2) radiation therapy which involves a high energy rays or particles to destroy the cancer cells. Systemic treatment includes the use of cytotoxic, hormonal, and immunotherapy which is usually used in the adjuvant, neoadjuvant, and metastatic settings. While these systemic agents are active and effective in the majority of breast cancers, after a variable period of time, progression occurs in some cases. Resistance to therapeutic drugs in breast cancer is multi-factorial. It may cause disturbances in the apoptotic machinery, p-glycoprotein and the multidrug resistance protein family, HER-2/neu gene amplification and protein expression, along with the expression of additional members of the epithelial growth factor receptor family, DNA ploidy, p53 gene mutations, cyclin E and p27 dysregulation that cumulatively drive the development of cancer stem cells [87].

Previous genome-wide association studies have identified a common risk variant at the *TERT-CLPTM1L* locus on chromosome 5p15 (odds ratio (OR) = 1.25, *p* = 1.1 × 10^−9^), which was present at greater frequency in African Americans than in women of European ancestry, and was significantly associated with triple negative breast cancer in younger women (aged < 50 years; OR = 1.48, *p* = 1.9 × 10^−9^) [7]. A series of scientific reports have linked breast cancer drug resistance to disturbed or over-expression of pro-survival stress genes including Bcl-1, Mcl-1 and other BH3 family members [88]. This is confirmed from the observation that BH3 mimetic ABT-737 can sensitize BH3 protein over-expressing breast cancer cells [89]. Other reports have also linked disturbed proteasome signaling to breast cancer chemo and radio resistance [90]. These findings become particularly relevant as proteasome inhibitor bortezomib treatment causes suppression of MDRs leading to induction of apoptosis in breast tumor models [91]. Recent collaborative studies by our group called “Getting to Know Cancer” have demonstrated that inherent resistant traits of breast cancer are associated with malfunctioning of the apoptosis signaling. Targeting the different pathways that either induce apoptosis or suppress the pro-survival signaling is expected to resulting in overcoming drug resistance to various therapies [92].

## 4. Conclusions

Collectively, this review paper provides considerable evidence that breast cancer mortality rate is significantly high among minority African American women population compared to White women in the United States [93,94,95,96]. For instance, a 2013 report indicated that the relative 5 year breast cancers survival rate was 78% among Black women and 90% among White women between 2002 and 2008 [97]. This high mortality rate in breast cancer among minorities may be attributed to lack of medical coverage, barriers to early detection and screening, more advanced stage of disease at diagnosis among minorities, and unequal access to improvements in cancer treatment [70,71,72]. Many African American women have frequent unknown or un-staged breast cancers than White women. These are scientific evidences that may explain the differences in breast cancer treatment and survival rate among African American and White women. With the current scientific knowledge and advance in early detection for breast cancer, there are great possibilities to reduce breast cancer incidence rate in the near future. Although there has been an improvement in early detection, diagnosis, and screening, many Black women are less likely to receive the same level of quality care compared with White women [20,21]. Therefore, new strategies and approaches are needed to promote breast cancer prevention, improve survival rate, reduce breast cancer mortality, and ultimately improve the health outcomes of racial/ethnic minorities.

## Figures and Tables

**Table 1:** Comparison of Cancer Incidence Rates between Non-Hispanic (NH) Blacks and Whites, United States, 2008–2012 [31].

Male					Female				
Cancer	NH Black Rate ^a^	NH White Rate ^a^	Absolute Difference ^b^	Rate Ratio ^c^	Cancer	NH Black Rate ^a^	NH White Rate ^a^	Absolute Difference ^b^	Rate Ratio ^c^
Kaposi sarcoma	1.7	0.5	1.2	3.57 ^d^	Kaposi sarcoma	0.2	<0.1	0.1	3.96 ^d^
Myeloma	14.8	7.0	7.8	2.11 ^d^	Myeloma	11.1	4.3	6.8	2.58 ^d^
Stomach	15.1	7.8	7.3	1.93 ^d^	Stomach	8.0	3.5	4.5	2.30 ^d^
Liver & IHB	16.5	9.3	7.2	1.77 ^d^	Liver & IHB	4.8	3.2	1.6	1.52 ^d^
Prostate	208.7	123.0	85.7	1.70 ^d^	Uterine cervix	10.0	7.1	2.9	1.41 ^d^
Larynx	9.3	6.3	3.0	1.48 ^d^	Pancreas	14.4	10.6	3.8	1.36 ^d^
Breast	2.0	1.4	0.6	1.45 ^d^	Esophagus	2.5	1.8	0.7	1.34 ^d^
Colon & rectum	60.3	47.4	12.9	1.27 ^d^	Colon & rectum	44.1	36.2	7.9	1.22 ^d^
Pancreas	17.2	14.0	3.2	1.23 ^d^	Kidney & renal pelvis	13.0	11.3	1.7	1.15 ^d^
Lung & bronchus	93.4	79.3	14.1	1.18 ^d^	Breast	124.3	128.1	−3.8	0.97 ^d^
Kidney & renal pelvis	24.2	21.8	2.4	1.11 ^d^	Uterine corpus	23.0	25.5	−2.5	0.90 ^d^
Hodgkin lymphoma	3.2	3.4	−0.2	0.95 ^d^	Hodgkin lymphoma	2.4	2.7	−0.3	0.88 ^d^
Esophagus	8.0	8.8	−0.8	0.90 ^d^	Lung & bronchus	51.4	58.7	−7.3	0.87 ^d^
Oral cavity & pharynx	15.3	18.1	−2.8	0.84 ^d^	Leukemia	8.6	10.7	−2.1	0.80 ^d^
Leukemia	13.2	17.7	−4.5	0.75 ^d^	Oral cavity & pharynx	5.2	6.7	−1.5	0.78 ^d^
Non-Hodgkin lymphoma	17.2	24.1	−6.9	0.71 ^d^	Ovary	9.6	12.4	−2.8	0.77 ^d^
Brain & ONS	4.9	8.8	−3.9	0.56 ^d^	Non-Hodgkin lymphoma	12.0	16.6	−4.6	0.72 ^d^
Urinary bladder	19.8	40.2	−20.4	0.49 ^d^	Urinary bladder	6.7	9.9	−3.2	0.68 ^d^
Thyroid	3.7	7.7	−4.0	0.48 ^d^	Thyroid	12.9	21.9	−9.0	0.59 ^d^
Testis	1.4	6.8	−5.4	0.21 ^d^	Brain & ONS	3.6	6.3	−2.7	0.58 ^d^
Melanoma of the skin	1.1	31.3	−30.2	0.04 ^d^	Melanoma of the skin	1.0	20.6	−19.6	0.05 ^d^
All sites	592.3	528.9	63.4	1.12 ^d^	All sites	408.1	436.2	−28.1	0.94 ^d^

IHB indicates intrahepatic bile duct; ONS, other nervous system; ^a^ Rates are per 100,000 and age adjusted to the 2000 US standard population; ^b^ The absolute difference is the rate in blacks minus the rate in whites; ^c^ The rate ratio is the unrounded rate in blacks divided by the unrounded rate in whites; ^d^ The rate ratio is significantly different from one (*p* < 0.05). Note: Sites are listed in descending order by rate ratio; Source: North American Association of Central Cancer Registries [31].
